# Transfusion practice in Central Norway – a regional cohort study in patients suffering from major haemorrhage

**DOI:** 10.1186/s12873-023-00918-3

**Published:** 2024-01-07

**Authors:** Marte Irene Skille Carlsen, Jostein Rødseth Brede, Christian Medby, Oddvar Uleberg

**Affiliations:** 1 Department of Anesthesiology and Intensive Care Medicine, St Olav’s University Hospital, Trondheim, Norway; 2 Department of Traumatology, St. Olav’s University Hospital, Trondheim, Norway; 3 Department of Emergency Medicine and Pre-hospital Services, St Olav’s University Hospital, Trondheim, Norway; 4 Department of Research and Development, Norwegian Air Ambulance Foundation, Oslo, Norway; 5 Norwegian Armed Forces Joint Medical Services, Sessvollmoen, Norway; 6 Department of Research and Development, Division of Emergencies and Critical Care, Oslo University Hospital, Oslo, Norway

**Keywords:** Haemorrhagic shock, Transfusion, Massive transfusion, Fluid administration, Plasma, Platelet, Red blood cell

## Abstract

**Background:**

In patients with major hemorrhage, balanced transfusions and limited crystalloid use is recommended in both civilian and military guidelines. This transfusion strategy is often applied in the non-trauma patient despite lack of supporting data. The aim of this study was to describe the current transfusion practice in patients with major hemorrhage of both traumatic and non-traumatic etiology in Central Norway, and discuss if transfusions are in accordance with appropriate massive transfusion protocols.

**Methods:**

In this retrospective observational cohort study, data from four hospitals in Central Norway was collected from 01.01.2017 to 31.12.2018. All adults (≥18 years) receiving massive transfusion (MT) and alive on admission were included. MT was defined as transfusion of ≥10 units of packed red blood cells (PRBC) within 24 hours, or ≥ 5 units of PRBC during the first 3 hours after admission to hospital. Clinical data was collected from the hospital blood bank registry (ProSang) and electronic patient charts (CareSuite PICIS). Patients undergoing cardiothoracic surgery or extracorporeal membrane oxygenation treatment were excluded.

**Results:**

A total of 174 patients were included in the study, of which 85.1% were non-trauma patients. Seventy-six per cent of all patients received plasma:PRBC in a ratio ≥ 1:2 (high ratio) and 59.2% of patients received platelets:PRBC in a ratio ≥ 1:2 (high ratio). 32.2% received a plasma:PRBC-ratio ≥ 1:1, and 23.6% platelet:PRBC-ratio ≥ 1:1. Median fluid infusion of crystalloids in all patients was 5750 mL. Thirty-seven per cent of all patients received tranexamic acid, 53.4% received calcium and fibrinogen concentrate was administered in 9.2%.

**Conclusions:**

Most patients had a non-traumatic etiology. The majority was transfused with high ratios of plasma:PRBC and platelet:PRBC, but not in accordance with the aim of the local protocol (1:1:1). Crystalloids were administered liberally for both trauma and non-trauma patients. There was a lower use of hemostatic adjuvants than recommended in the local transfusion protocol. Awareness to local protocol should be increased.

## Background

Major hemorrhage is a clinical emergency that occurs regularly in both medical and surgical patients [1]. It is a leading cause of potentially preventable deaths [2], and the second most common cause of trauma-related deaths [3]. During the last two decades, protocols for massive transfusion (MT) and balanced transfusions have been developed, particularly within the area of trauma-related major hemorrhage [4]. However, in Norway and presumably most western countries, the majority of patients who receive massive transfusion have a non-traumatic etiology, dominated by post-partum hemorrhage, gastrointestinal hemorrhage, ruptured aortic aneurism or perioperative hemorrhage [5–7].

The concepts of damage control surgery and damage control resuscitation (DCR) include restoring normal physiology by early hemorrhage control and mitigation of coagulopathy, acidosis and hypothermia through the balanced transfusion of packed red blood cells (PRBCs), plasma and platelets [8, 9] and reduced use of isotonic crystalloids as they have been shown to aggravate coagulopathy and increase mortality [10–12]. Oxygen depth and hypoperfusion due to extensive trauma causes severe endotheliopathy and glycocalyx shedding of heparans and thrombomodulin provoking endogenous anticoagulation and coagulopathy [13]. Several studies have shown that a higher plasma to PRBC ratio in MT, typically 1:2 or greater, is associated with better survival [14–16]. Both civilian and military authoritative guidelines, as well as our local protocol, recommend a ratio of 1:1:1 of PRBC, plasma and platelets [17, 18]. Hence, balanced transfusions with limited crystalloid use is regarded as best practice in management of major hemorrhage in trauma [19–21].

The DCR strategy is often transferred directly to the non-trauma setting [22]. Despite the lack of supporting data from non-trauma patients, this approach seems to be common practice [6, 23]. Hence, the aim of this study was to describe the current transfusion practice in patients with major hemorrhage of both traumatic and non-traumatic etiology in Central Norway and discuss if practice relates to available massive transfusion protocols.

## Methods

### Study design

A retrospective observational cohort study with data from four hospitals in Central Norway. The study follows the ‘Strengthening the reporting of observational studies in epidemiology’ (STROBE) recommendations for reporting of observational cohort studies [24].

### Study setting

Central Norway has an adult population of approximately 474,000 and comprises both rural and urban areas. Eight hospitals cover the region, of which seven are acute care hospitals (ACH) and one is a tertiary care hospital (TCH), St. Olav’s University Hospital (SOHO) in Trondheim. SOHO is also the regional trauma center. All hospitals admit acute medical and surgical patients. The four hospitals that use the electronic patient record CareSuite PICIS (PICIS, Wakefield, MA), were included in the study. This included SOHO as well as the hospitals in Orkdal, Levanger and Namsos. All hospitals have a blood bank with PRBC and frozen plasma (Octaplasma), but the ACHs have limited units of platelets. Plasma must be thawed before dispatch. Whole blood was not available in the study period.

### Data collection

**Fig. 1:**
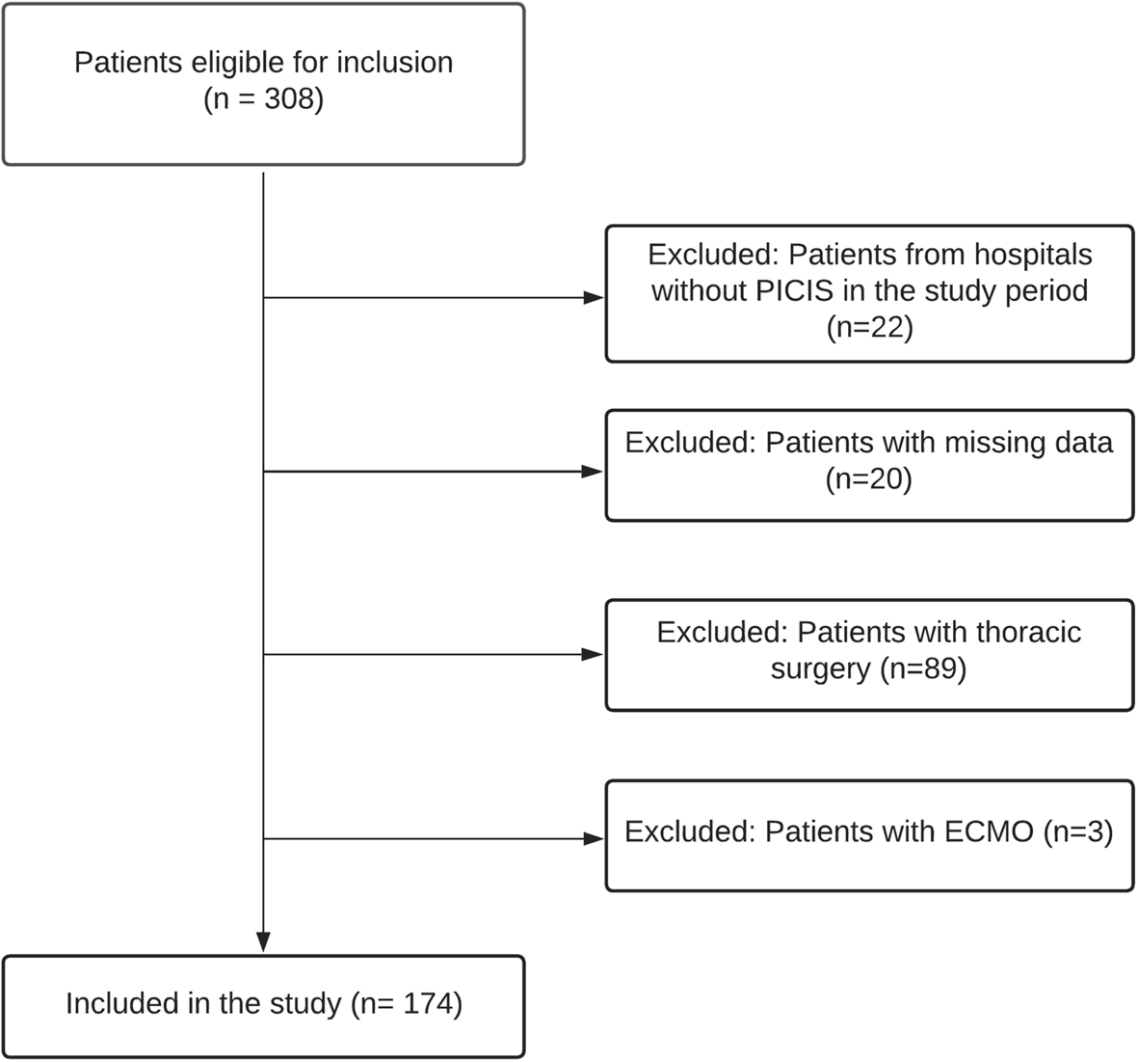
Flowchart of inclusion process. The flowchart gives an overview of the inclusion process. PICIS indicates electronic patient chart. ProSang indicates hospital blood bank registry

All adults (≥18 years) receiving MT at the included hospitals in the two-year period from 01 January 2017 to 31 December 2018 who were alive on admission, were eligible for inclusion. Transfused patients were identified using the hospital blood bank registry (ProSang). Data was collected from the same population as previously studied by Godø et al. [5], however different inclusion and exclusion criteria were used. Patients were excluded if 1) data from the electronic patient chart (CareSuite PICIS) or from the hospital blood bank registry ProSang (CSAM Health Group, Oslo, Norway) were missing; 2) patients were undergoing thoracic surgery with the use of cardiopulmonary by-pass intervention; and if 3) patients were undergoing extracorporeal membrane oxygenation (ECMO) treatment, as patients in group 2) and 3) often require blood products due to the mechanical treatment (Fig. 1) [25]. In case of conflicting data between record systems, Caresuite PICIS was used, as it provides the most granular data. Information from ProSang, patient records and PICIS were manually retrieved. MT was defined as transfusion of ≥10 units of PRBC within 24 hours [2] or ≥ 5 units of PRBC during the first 3 hours after admission to hospital [21], similar to the definitions used in our regional MT protocol (MTP) for trauma. The trauma protocol is valid for all hospitals in the region (Table 1) and has remained unchanged since before the study period.

**Table 1:** Massive transfusion protocol in Central Norway

Criteria for initiation of transfusion protocol	Suspected severe bleeding and hypotension (systolic blood pressure < 90 mmHg) or other signs of shock.
**Transfusion protocol**	750 mL packed red blood cell concentrate + 600 mL of Octaplasma [1] + 250 mL platelet concentrate (repeat as needed) Fibrinogen concentrate (e.g. Riastap or Fibryga) 2–4 g (repeat as needed).
**Adjuncts included in protocol**	Tranexamic acid 1 g (bolus) + tranexamic acid 1 g (infusion over 8 hours) Calcium gluconate (20–40 mL, repeat as needed for plasma Ca^2+^ concentration < 1.1 mmol/L) Consider specific drugs for reversal of anticoagulants (e.g. prothrombin complex concentrate for Warfarin or Idarusizumab for Dabigatran)

^*1*^*Octaplasma is a commercially available pooled, pathogen inactivated frozen plasma product (Octapharma). It is the standard product used for only plasma substitution in Norway*

The initiation of a MT was defined as the time for the first PRBC order that triggered MT. Additional variables were recorded in the following 25-hour period, from 1 hour before start of MT to 24 hours after start of MT. The patient records were reviewed for demographics, transfusion indication, American Society of Anaesthesiologists (ASA) score, hemorrhage etiology and use of anticoagulation and hemostatic drugs. Both electronic and written patient records were reviewed.

### Transfusion ratio calculation

In our MTP, blood products are dispatched from the blood bank as packs containing 3 units of PRBC, 3 units of frozen, pathogen inactivated pooled plasma (Octaplasma, Octapharma), and one unit of platelets (containing approximately 3 units of single donor platelets from donor apheresis or from multiple whole blood donations). The packs are designed to provide balanced transfusion, each containing 3 units of whole blood equivalents (750 mL PRBC, 600 mL plasma and 250 mL platelets). These volumes were used to calculate transfusion ratios; hence, 750 ml PRBC to 250 ml platelets equals a 1:1 ratio, similarly 750 ml PRBC to 600 ml plasma.

The number of units administered during the 25-hour period was summarized and ratios for plasma:PRBC and platelets:PRBC calculated to evaluate transfusion balance. Administration of more than one unit of plasma or platelet per 2 units PRBC (> 1:2) was considered high ratio, whilst less than one unit of plasma per 2 units of PRBC was considered low ratio, in accordance with survival benefits demonstrated in prospective studies [26].

### Statistical analysis

Descriptive characteristics are presented as means with standard deviation or median with interquartile range, as appropriate, and as absolute numbers and percentages. No statistical comparisons between groups were performed. Data analysis was performed using SPSS statistical software (IBM Corporation. SPSS Statistics for Windows, Version 27.0, IBM Corporation, Armonk, NY, USA).

### Ethics

The study was performed in accordance with the Helsinki declaration for medical research involving human subjects. The Regional committees for medical and health research ethics committee of Central Norway (REK - Midt) was informed about the study, and deemed the study a clinical quality study not needing formal Regional Ethics Committee (REC) approval (reference number 2019/793/REC Central), which waived the need for patient consent.

## Results

### General results

Patient characteristics are described in Table 2. One-hundred-and-seventy-four patients were included in the study, whereas 94% (*n* = 163) of the patients were treated at the TCH and 6% (*n* = 11) were treated at the ACHs.

**Table 2:** Baseline characteristics of patients (*n* = 174)

Characteristics	
**Male, n (%)**	104 (59.8)
**Age, years mean (range)**	62.1 (21–93)
**Male mean**	65.2
**Female mean**	57.2
**ASA score**, **n (%)**	
** 1**	1 (0.6)
**2**	26 (14.9)
**3**	68 (39.1)
**4**	63 (36.2)
**5**	14 (8.0)
**Missing**	2 (1.1)
**Anticoagulation therapy n (%)**	68 (39.1)
**Traumatic aetiology n (%)**	26 (14.9)
**Haemorrhagic aetiology n (%)**	
**Colorectal surgery**	62 (35.6)
**Vascular surgical**	31 (17.8)
**Gynaecology**	29 (16.7)
**Trauma**	26 (14.9)
**Urology**	11 (6.3)
**Orthopaedic**	7 (4.0)
**Internal medicine**	4 (2.3)
**Neurosurgery, plastic surgery, other**	4 (2.3)

*ASA score: American Society of Anesthesiologist score*

Two patients were eligible due to 10 PRBC in 24 hours, the remainder were all eligible through the five PRBC within 3 hours criterion.

Three quarters of all patients received plasma:PRBC in a ratio ≥ 1:2 (high ratio), and 59.2% of patients received platelets:PRBC in ≥1:2-ratio (high ratio) (Table 3). Within the high ratio group, 32.2% received a plasma:PRBC-ratio ≥ 1:1, and 23.6% platelet:PRBC-ratio ≥ 1:1. This would represent an optimal adherence to local protocol. A median amount of 5750 ml of crystalloids were administered. Sixty-three patients (36.2%) received more than seven liters and 25 patients (14.3%) received more than 10 liters of crystalloids. When comparing administration of TXA between the trauma and non-trauma group, we observed a higher proportion of trauma patients receiving TXA (57.7% versus 33.1%) (Table 2).

**Table 3:** Transfusion volumes, transfusion ratios and use of hemostatic agents within the whole population, trauma- and non-trauma patients administered during the 25 hour registration period

	All	Trauma	Non-Trauma
**Total n (%)**	174 (100)	26 (14.9)	148 (85.1)
**Fluid infusion (mL)**			
**PRBC median (IQR)**	2000 (1125)	2000 (1063)	2000 (1250)
**Plasma median (IQR)**	1200 (1000)	1300 (1050)	1200 (1000)
Platelets median (IQR)	250 (500)	250 (313)	250 (500)
**Crystalloids median (IQR)**	5750 (4181)	5950 (3713)	5700 (4355)
**Plasma:PRBC-ratio ≥ 1:2 n (%)**	133 (76.4)	21 (80.8)	112 (75.7)
**Platelets:PRBC-ratio ≥ 1:2 n (%)**	103 (59.2)	16 (61.5)	87 (58.8)
**Medications used**			
**TXA (gram) median (IQR)**	1.0 (1.0)	1.0 (1.0)	1.0 (0.8)
**Patients received TXA n (%)**	64 (36.8)	15 (57.7)	49 (33.1)
**Calcium (mmol) median (IQR)**	8.0 (5.0)	5.0 (0)	12.5 (15)
**Patients received calcium n (%)**	93 (53.4)	12 (46.2)	81 (54.7)
**Fibrinogen concentrate (gram) median (IQR)**	1.5 (1.0)	1.5 (0)	1.75 (1.0)
**Patients received fibrinogen concentrate n (%)**	16 (9.2)	4 (15.4)	12 (8.1)

*IQR: Interquartile Range, PRBC: Packed Red Blood Cells TXA: Tranexamic Acid*

## Discussion

We found a high ratio of balanced transfusions between plasma and PRBC, but a lower degree between platelets and PRBC. The latter may be due to low availability of platelets at the ACHs. By volume, our plasma:PRBC ratios are lower, as Octaplasma is standardized to 200 mL, while the volume of single donor fresh frozen plasma (FFP) would range from 200 mL to 250 mL This reflects national guidelines, but makes comparisons to FFP:PRBC studies challenging.

There were no differences in transfusion ratios between the trauma and non-trauma group. One might have expected the trauma group to have superior transfusion ratios, as this is explicitly described in the local MTP. This may reflect a change in practice where clinicians have adopted high plasma to PRBC-ratio strategies, without distinguishing between hemorrhage etiologies. The PROPPR trial demonstrated reduced mortality from exsanguination within the first 24 hours for trauma patients receiving 1:1:1-ratio vs 1:1:2-ratio [26]. However, the reduction in 24-hour or 30-day mortality was not significant compared to a 1:1:2-ratio. For the diverse group of non-trauma patients, the uncertainty might be even greater [27].

We observed a liberal use of crystalloids (Table 3) where 36% received more than seven liters. There are plausible explanations beyond poor protocol adherence, such as crystalloid used as volume replacement in absence of bleeding or prior to onset of major hemorrhage, as mixture solution for medications. There is also a historical aspect where crystalloids were important in resuscitation of hypovolemia. This may influence resuscitation of bleeding patients, despite well-documented complications such as dilution coagulopathy, multi organ failure, abdominal compartment syndrome and adult respiratory distress syndrome [28, 29].

In the trauma MTP, TXA is recommended if administered within 3 hours of injury [30]. Some patients may have arrived later than 3 hours after injury, however we still assume TXA was indicated for most trauma patients. In non-trauma hemorrhage both TXA and fibrinogen concentrate are mentioned as treatment measures [31–33]. S-Calcium are easily monitored through arterial blood gas values, as severe hypocalcemia is common in MT [34]. Hence, the use of adjuvant medication was low, given our local transfusion protocol’s clear recommendations.

MTP improves outcomes in hemorrhaging trauma patients [35–37]. Best practice for non-trauma patients are unknown, [38, 39] but our study indicates that a similar transfusion strategy is applied to all bleeding patients regardless of diagnosis. Whether a different and tailored treatment should be applied to the non-trauma group needs to be addressed in future studies.

### Limitations

There are several limitations. The small sample size does not allow for a description of subgroups, nor statistical comparisons between groups. Data was manually registered into the electronic patient charts, which allows for erroneous input. Further, unused blood products returned to the blood bank also relies on manual registration and would allow for error where large quantities were administered.

Blood product ratios may have varied during the period of each transfusion incidence, as only the final ratios were captured in the data collection. However, as most patients fell within the three-hour definition, we expect that products were dispatched as transfusion packs. This is not possible to determine from the electronic patient charts. Further, viscoelastic tests and coagulopathy tests are not routine, and therefore not provided in this study. Parameters reflecting bleeding extension was not collected as this was a study mainly using data from the blood bank registry. The data is from one region in Norway and the generalizability to other Norwegian or Scandinavian hospitals is unknown.

## Conclusion

Most patients that received massive transfusion had a non-traumatic etiology. The majority was transfused with high ratios of plasma:PRBC and platelet:PRBC, but not in accordance with the aim of the local protocol (1:1:1). Crystalloids were administered liberally for both trauma and non-trauma patients. There was a lower use of hemostatic adjuvants than recommended in the local transfusion protocol. Awareness to local protocol should be increased.

## Data Availability

Data is available upon reasonable request.

## Abbreviations

ACH: Acute care Hospital
ASA: American Society of Anaesthesiologists physical status
DCR: Damage Control Resuscitation
ECMO: Extracorporeal Membrane Oxygenation
MT: Massive transfusion
MTP: Massive Transfusion Protocol
PRBC: Packed Red Blood Cells
REC: Regional Ethics Committee
SOHO: St. Olav’s University hospital
STROBE: Strengthening the reporting of observational studies in epidemiology
TCH: Tertiary Care Hospital
TXA: Tranexamic acid
